# Second-line drug-resistant TB and associated risk factors in Karakalpakstan, Uzbekistan

**DOI:** 10.5588/ijtldopen.24.0351

**Published:** 2024-09-01

**Authors:** S. Moe, I. Azamat, S. Allamuratova, M. Oluya, A. Khristusev, M.L. Rekart, K. Mamitova, G. Bidwell, C. Gomez-Restrepo, B. Kalmuratov, Z. Tigay, N. Parpieva, K. Safaev, N. Sitali, D. Gomez, A. Mikhail, A. Sinha

**Affiliations:** ^1^ Médecins Sans Frontières (MSF), Nukus, Uzbekistan;; ^2^ MSF, Tashkent, Uzbekistan;; ^3^ Republican Center of Tuberculosis and Pulmonology, Nukus, Uzbekistan;; ^4^ Republican Specialised Scientific and Practical Medical Center of Tuberculosis and Pulmonology, Tashkent, Uzbekistan;; ^5^ MSF, Berlin, Germany;; ^6^ MSF, Amsterdam, Netherlands;; ^7^ MSF, London, UK.

**Keywords:** epidemiology, second-line drug-resistant TB, Group-A drug-resistant TB, risk factors

## Abstract

**BACKGROUND:**

Drug-resistant TB (DR-TB) remains a major public health threat. In 2022, Uzbekistan reported 2,117 cases of DR-TB, with 69% tested for fluoroquinolone resistance. Limited information is available on the prevalence of resistance to bedaquiline, linezolid, and fluoroquinolone, which are key components of the all-oral treatment regimen for rifampicin-resistant TB in Uzbekistan.

**METHODS:**

A retrospective study was conducted using extensive programmatic data from 2019 to 2023 in Uzbekistan. We assessed second-line drug-resistant TB (SLDR-TB) rates using phenotypic drug susceptibility testing (pDST). Demographic and clinical characteristics associated with SLDR-TB were analysed using multivariable logistic regression models based on the Allen-Cady approach.

**RESULTS:**

In total, 2,405 patients with TB who had undergone pDST were included (median age 40 years, 47% female). The overall SLDR-TB resistance rate was 24% (95% CI 22–26). Prevalence of resistance to bedaquiline, linezolid, moxifloxacin, levofloxacin, and amikacin were respectively 3.1%, 0.8%, 15%, 13%, and 12%. Risk factors for SLDR-TB were resistance to rifampicin and/or isoniazid, exposure to clofazimine, retreatment status, contact with drug-susceptible TB case or DR-TB case, and diabetes.

**CONCLUSIONS:**

The high prevalence of SLDR-TB is of major concern, emphasising the need for baseline pDST in RR-TB treatment. Identified risk factors can aid early detection of at-risk individuals and inform clinical practice.

## RÉSUMÉ

**CONTEXTE:**

La TB résistante aux médicaments (DR-TB) reste une menace majeure pour la santé publique. En 2022, l'Ouzbékistan a signalé 2 117 cas de DR-TB, dont 69% ont été testés pour la résistance aux fluoroquinolones. Les informations sur la prévalence de la résistance à la bédaquiline, au linézolide et aux fluoroquinolones, qui sont des composants clés du traitement entièrement oral de la TB résistante à la rifampicine en Ouzbékistan, sont limitées.

**MÉTHODES:**

Une étude rétrospective a été menée en utilisant des données programmatiques exhaustives de 2019 à 2023 en Ouzbékistan. Nous avons évalué les taux de TB résistante aux médicaments de deuxième ligne (SLDR-TB, pour l’anglais, « second-line drug-resistant TB ») en utilisant des tests de sensibilité phénotypique aux médicaments (pDST). Les caractéristiques démographiques et cliniques associées à la SLDR-TB ont été analysées à l'aide de modèles de régression logistique multivariés basés sur l'approche Allen-Cady.

**RÉSULTATS:**

Au total, 2 405 patients atteints de TB ayant subi un pDST ont été inclus (âge médian de 40 ans, 47% de femmes). Le taux global de résistance à la SLDR-TB était de 24% (CI à 95% 22–26). La prévalence de la résistance à la bédaquiline, au linézolide, à la moxifloxacine, à la lévofloxacine et à l'amikacine était respectivement de 3,1%, 0,8%, 15%, 13% et 12%. Les facteurs de risque de SLDR-TB comprenaient la résistance à la rifampicine et/ou à l'isoniazide, l'exposition à la clofazimine, le statut de retraitement, le contact avec un cas de TB sensible aux médicaments ou de DR-TB, et le diabète.

**CONCLUSIONS:**

La prévalence élevée de la SLDR-TB est une source de préoccupation majeure, soulignant la nécessité de réaliser des pDST de base dans le traitement de la TB résistante à la rifampicine. Les facteurs de risque identifiés peuvent aider à la détection précoce des individus à risque et à informer la pratique clinique.

Drug-resistant TB (DR-TB), particularly multidrug-resistant/rifampicin-resistant TB (MDR/RR-TB), pre-extensively drug-resistant TB (pre-XDR-TB), and extensively drug-resistant TB (XDR-TB), poses a growing global public health threat.^1^ In 2022, the WHO reported 153,274 cases of MDR/RR-TB and 27,075 cases of pre-XDR-TB or XDR-TB. Surprisingly, only 54% of these cases were tested for fluoroquinolone (FQ) resistance.^2^ Bedaquiline (BDQ), linezolid (LZD), and moxifloxacin (MFX) are key components of the all-oral treatment regimen for MDR/RR-TB and pre-XDR-TB.^1^ A survey conducted in Europe revealed limited capacity for testing the susceptibility of *Mycobacterium tuberculosis* (MTB) to these new or repurposed drugs,³ and information on the country-specific prevalence of resistance to these drugs is even more scarce.

Studies conducted before the 2021 WHO definition of pre-XDR-TB and XDR-TB revealed that the proportion of pre-XDR-TB and XDR-TB among patients with MDR/RR-TB is higher in high-burden countries such as Russia (40.4% pre-XDR-TB, 19.2% XDR-TB),^4^ India (32.4% pre-XDR-TB, 4.7% XDR-TB),^5^ South Africa (22.3% pre-XDR-TB),^6^ Myanmar (26.9% pre-XDR-TB, 13.5% XDR-TB),^7^ and Ukraine (55% pre-XDR or XDR-TB)^8^ than in low-burden countries such as Taiwan (14.8% pre-XDR-TB, 1.7% XDR-TB),^9^ and Brazil (9.19% pre-XDR-TB, 4.59% XDR-TB).^10^

In 2021, the WHO updated the definitions of pre-XDR-TB and XDR-TB, emphasising their seriousness and the need for rapid molecular tests to detect FQ resistance and phenotypic drug susceptibility testing (pDST) for BDQ and LZD. A systematic review study reported the pooled percentage of FQ-resistant cases among patients with MDR-TB as 27%. The study also estimated the pooled proportion of resistance to BDQ as 5%, LZD as 4%, and clofazimine (CFZ) as 4% among patients with MDR-TB.^11^

A study from India reported the prevalence of FQ resistance among MDR-TB strains at an alarming level of 73.6%.^12^ Similarly, a study from China of MDR-TB isolates reported a prevalence of 73.2% for FQ resistance, 7.1% for LZD resistance, and 2.4% for BDQ resistance.^13^ A nationwide prevalence survey for MDR-TB conducted in Uzbekistan in 2010–2011 reported a prevalence of 12.5% (40/319) resistance to any FQ among patients with MDR-TB,^14^ which is considerably lower than the global average of 20.1% reported by the WHO.^15^ Limited information is available on the epidemiology of second-line drug-resistant TB (SLDR-TB) and Group A drug-resistant TB (GADR-TB) in Karakalpakstan and Uzbekistan in general.

BDQ was introduced as a standard of care treatment in Karakalpakstan in 2015. Since then, a growing number of primary resistance^16^ and acquired resistance^17^ to BDQ have been reported in the region. Our study’s primary objective was to bridge the information gap by conducting a retrospective study to comprehend the trends and resistance patterns of SLDR-TB in Karakalpakstan. Several demographic and clinical characteristics have been reported to be associated with SLDR-TB.^18^^–22^ Therefore, we aimed to leverage extensive programmatic data and gain deeper insights into the risk factors related to SLDR-TB and GADR-TB in this study setting.

## METHODS

### Study setting

Karakalpakstan, an autonomous republic within Uzbekistan with a total population of 1.9 million in 2022 and is divided into 16 districts with Nukus city as the capital.^23^ Médecins Sans Frontières (MSF) has collaborated closely with the Ministry of Health (MoH) in Karakalpakstan for over two decades, focusing on TB laboratory diagnosis, clinical management, and DR-TB surveillance.

We conducted a retrospective study using data collected between January 2019 and August 2023 from TB patients who underwent pDST for second-line drugs in Karakalpakstan.

### Laboratory testing algorithm

Xpert Ultra (Cepheid, Sunnyvale, CA, USA) testing served as a screening test for all TB-suspected patients. Positive results from Ultra (Cepheid; Sunnyvale, CA, USA) or GenoType MTBDR*plus* (Hain Lifescience, Nehren, Germany), including samples from follow-up patients who underwent pDST, were also included. This pDST covered MFX, levofloxacin (LFX), BDQ, CFZ, LZD, and amikacin (AMK), following national TB treatment guidelines (Supplementary Figure S1).

### Phenotypic drug susceptibility testing

We conducted pDST for LFX, MFX, and LZD using BD BACTEC^TM^ MGIT^TM^ (Mycobacterial Growth Indicator Tube) SIRE kit with the 960 system (BD, Franklin Lakes, NJ, USA) and EpiCenter software equipped with the TB eXiST module (Becton and Dickinson, Diagnostic Systems, Sparks, MD, USA). Critical concentrations (CCs) were as follows: 1.0 mg/L for LFX, LZD, and AMK; 0.25 mg/L and 0.5 mg/L for MFX in 2019; and 0.25 mg/L and 1.0 mg/L for MFX from 2020 onwards. CFZ initially had a CC of 2.5 mg/L due to a laboratory error (January 2019 to May 2022), later corrected to 1.0 mg/L from June 2022. BDQ fumarate (obtained from the NIH HIV Reagent Program) was tested at a CC of 1.0 mg/L.^24^

### Quality control

Batch testing was conducted for each new drug using H37Rv as a susceptible strain reference and the respective resistant strains provided by the WHO Supranational Reference Laboratory (SRL) for TB in Gauting, Germany. For every pDST performed, an H37Rv reference sample and drug-free growth control were included. The SRL Gauting provided annual quality assurance for molecular tests and pDST of MTB. Bacterial isolates’ susceptibility was tested using drug powders manufactured by Jansen Pharmaceuticals (Beerse, Belgium). pDST results were determined when the drug-free growth control tube displayed more than 400 growth units (GU) and was interpreted as follows: ‘susceptible’ for no growth in the drug vial, “intermediate” for 1–399 GU, and ‘resistant’ for >400 GU.

### Data collection

Each sample submitted to the laboratory received a unique laboratory number and patient identifier. Testing data, including culture, pDST, Ultra, and Hain tests, were stored in the BD EpiCenter Microbiology Data Management System (Becton and Dickinson). Relevant variables—such as laboratory numbers, patient identifiers, birthdates, sex, sample collection dates, and test results—were extracted from the database.

Confirmed patients with RR-TB starting MDR/RR-TB treatment received a unique treatment ID, recorded in a local epidemiological (EPI) database jointly managed by MSF and MoH Karakalpakstan. The EPI database captured current and previous treatment history (including exposure to second-line), demographics, comorbidities (including HIV status, diabetes mellitus [DM], psychiatric disorders, hepatic and renal diseases, and cardiovascular disease), employment status, imprisonment history, migration history, contact with patients with DS-TB and DR-TB, chest X-ray findings, and alcohol and tobacco use status.

### Definitions

GADR-TB was defined as patients with TB resistant to BDQ, LZD, MFX, or LFX using pDST. SLDR-TB was defined as patients with TB resistant to BDQ, MFX, LFX, LZD, CFZ, or AMK by pDST.

### Data analysis

The unique patient identifier was used to differentiate pDST results across different visits, while the treatment ID was used to link between laboratory and EPI databases for risk factor analysis. SLDR-TB and GADR-TB rates were analysed using pDST data from the laboratory database, which were presented as percentages.

Risk factor analysis included patients with available clinical and treatment history in the EPI database. We constructed multivariable logistic regression models using the Allen-Cady approach for variable selection to identify associations between SLDR-TB and study variables.^25^ Variables included were age category, sex, TB case classification, diagnostic smear, chest X-ray findings, DM, HIV status, and second-line drug exposure as the backbone of the model based on existing literature.^18^^–22^ The remaining variables were added one by one, and only variables with a *P*-value lower than 0.05 remained in the final model. We performed a sensitivity analysis using the least absolute shrinkage and selection operator (LASSO)^26^ to assess model variation and calculated a 95% confidence interval. Data manipulation and analysis were performed using R v4.0.2 (R Computing, Vienna, Austria). This study met MSF’s Independent Ethical Review Board exemption criteria as a retrospective review of routinely collected data following Uzbekistan’s National TB Programme guidelines.

## RESULTS

**Figure 1.**
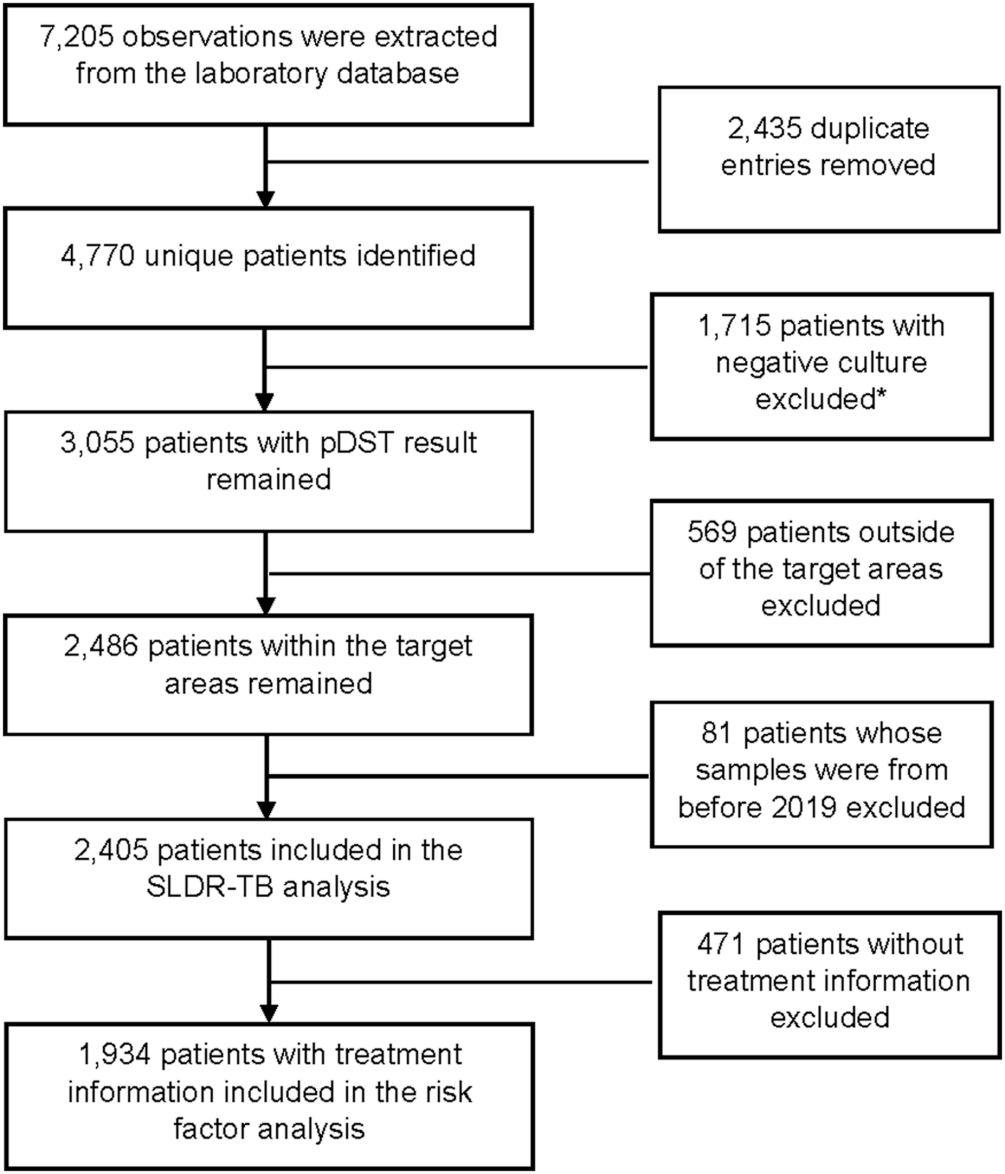
Flow chart of the study population. *The rifampicin and isoniazid results from genotypic testing GenoType MTBDR*plus* (Hain Lifescience, Nehren, Germany); and Xpert Ultra tests (Cepheid, Sunnyvale, CA, USA) were retained for the corresponding patients. pDST = phenotypic drug susceptibility testing, SLDR-TB = second-line drug-resistant TB.

In total, 7,205 observations were extracted from the laboratory database, including 4,770 unique patients, 1,715 patients with negative culture results, 569 patients outside the target area, and 81 patients for whom samples were collected before 2019 were excluded. The final study population included 2,405 patients for SLDR-TB analysis and 1,934 patients for risk factor analysis (Figure 1).

The median age of patients included in the SLDR-TB analysis was 40 years (interquartile range [IQR] 28–59), 47.0% were female, and 82.3% had resistance to rifampicin (RIF), isoniazid (INH), or both (Table 1). Characteristics of patients included in risk factors analysis were as follows: median age 40 years (IQR 28–58), 47.0% female, 2.0% healthcare workers, 16.0% had contact history with DR-TB, 13.0% with DS-TB exposure, 3.0% had CFZ exposure, and 36.0%, 37.0%, and 8.0% had exposure history to first-line, second-line, and Group A TB drug, respectively. Clinically, 92.0% had pulmonary TB, 44.0% had chest X-ray cavities, 45.0% had smear 1+ and above, and 14.0% had DM (Supplementary Table S1).

**Table 1.** Baseline characteristics of the study population included in the second-line drug-resistant analysis.

Characteristic	(*n* = 2,405)
	*n* (%)
Age, years, median [IQR]	40 [28–59]
Female sex	1,134 (47)
Sample collection year	
2019	561 (23)
2020	683 (28)
2021	442 (18)
2022	446 (19)
2023	273 (11)
FLDR-TB	
Resistant to RIF and INH	870 (55)
Resistant to RIF	20 (1.3)
Resistant to INH	419 (26)
Susceptible to RIF and INH	283 (18)
Unknown, *n*	813

IQR = interquartile range; FLDR-TB = first-line drug-resistant TB; RIF = rifampicin; INH = isoniazid.

Overall resistance rates were 16.0% (95% confidence interval [CI] 14–17) for GADR-TB and 24.0% (95% CI 22–26) for SLDR-TB. These rates were considerably higher in 2019 when the pDST for BDQ, LZD, and CFZ was newly introduced, and testing was mainly based on clinical considerations. The rates of GADR-TB and SLDR-TB decreased in 2020 when the testing algorithm was revised to include pDST testing for second-line drugs for all RIF-resistant samples, stabilising at around 16.0% for GADR-TB and 25.0% for SLDR-TB in 2021–2023 (Table 2).

**Table 2.** Distribution of phenotypic drug susceptibility testing for bedaquiline, linezolid, moxifloxacin, levofloxacin, amikacin, GADR-TB, FLDR-TB and SLDR-TB by year.

Variable	Overall		2019	2020	2021	2022	2023
	(*n* = 2,405)	Overall	(*n* = 561)	(*n* = 683)	(*n* = 442)	(*n* = 446)	(*n* = 273)
	*n* (%)	(95% CI)	*n* (%)	*n* (%)	*n* (%)	*n* (%)	*n* (%)
Bedaquiline							
Resistant	61 (3.1)	2.4‒4.0	6 (4.5)	18 (2.6)	14 (3.2)	9 (2.0)	14 (5.1)
Susceptible	1,813 (92)	90‒93	120 (91)	636 (93)	412 (93)	412 (92)	233 (85)
Indeterminate	101 (5.1)	4.2‒6.2	6 (4.5)	28 (4.1)	16 (3.6)	25 (5.6)	26 (9.5)
Unknown	430		429	1	0	0	0
Linezolid							
Resistant	15 (0.8)	0.44‒1.3	0 (0)	0 (0)	4 (0.9)	8 (1.8)	3 (1.1)
Susceptible	1,857 (94)	93‒95	126 (95)	654 (96)	425 (96)	409 (92)	243 (89)
Indeterminate	103 (5.2)	4.3‒6.3	6 (4.5)	28 (4.1)	13 (2.9)	29 (6.5)	27 (9.9)
Unknown	430		429	1	0	0	0
Moxifloxacin							
Resistant	312 (15)	13‒16	82 (30)	62 (9.1)	54 (12)	69 (15)	45 (16)
Susceptible	1,727 (82)	80‒83	170 (63)	605 (89)	381 (86)	362 (81)	209 (77)
Indeterminate	77 (3.6)	2.9‒4.6	20 (7.4)	16 (2.3)	7 (1.6)	15 (3.4)	19 (7.0)
Unknown	289		289	0	0	0	0
Levofloxacin							
Resistant	139 (13)	NA	0 (NA)	0 (0)	43 (11)	61 (14)	35 (13)
Susceptible	921 (83)	NA	0 (NA)	1 (50)	337 (87)	367 (82)	216 (79)
Indeterminate	47 (4.2)	NA	0 (NA)	1 (50)	6 (1.6)	18 (4.0)	22 (8.1)
Unknown	1,298		561	681	56	0	0
Fluoroquinolone							
Resistant	314 (15)	13‒16	82 (30)	62 (9.1)	56 (13)	69 (15)	45 (16)
Susceptible	1,726 (82)	80‒83	170 (63)	605 (89)	379 (86)	363 (81)	209 (77)
Indeterminate	76 (3.6)	2.9‒4.5	20 (7.4)	16 (2.3)	7 (1.6)	14 (3.1)	19 (7.0)
Unknown	289		289	0	0	0	0
Clofazimine							
Resistant	25 (1.3)	0.84‒1.9	4 (3.0)	4 (0.6)	1 (0.2)	3 (0.7)	13 (4.8)
Susceptible	1,865 (94)	93‒95	122 (92)	657 (96)	428 (97)	422 (95)	236 (86)
Indeterminate	84 (4.3)	3.4‒5.3	6 (4.5)	20 (2.9)	13 (2.9)	21 (4.7)	24 (8.8)
Unknown	431		429	2	0	0	0
Amikacin							
Resistant	248 (12)	11‒14	30 (14)	76 (11)	55 (12)	58 (13)	29 (11)
Susceptible	1,730 (84)	82‒85	178 (80)	595 (87)	380 (86)	361 (81)	216 (79)
Indeterminate	86 (4.2)	3.4‒5.1	14 (6.3)	12 (1.8)	7 (1.6)	25 (5.6)	28 (10)
Unknown	341		339	0	0	2	0
GADR-TB status							
Resistant	333 (16)	14‒17	83 (30)	66 (9.7)	65 (15)	72 (16)	47 (17)
Susceptible	1,717 (81)	79‒83	177 (65)	602 (88)	371 (84)	360 (81)	207 (76)
Indeterminate	67 (3.2)	2.5‒4.0	13 (4.8)	15 (2.2)	6 (1.4)	14 (3.1)	19 (7.0)
Unknown	288		288	0	0	0	0
SLDR-TB status							
Resistant	505 (24)	22‒26	105 (38)	117 (17)	105 (24)	110 (25)	68 (25)
Susceptible	1,558 (74)	72‒75	159 (58)	557 (82)	332 (75)	323 (72)	187 (68)
Indeterminate	54 (2.6)	1.9‒3.3	9 (3.3)	9 (1.3)	5 (1.1)	13 (2.9)	18 (6.6)
Unknown	288		288	0	0	0	0
FLDR-TB status							
Resistant to RIF and INH	870 (55)	52‒57	359 (73)	295 (45)	162 (65)	34 (26)	20 (29)
Resistant to RIF	20 (1.3)	0.79‒2.0	8 (1.6)	7 (1.1)	3 (1.2)	1 (0.8)	1 (1.5)
Resistant to INH	419 (26)	24‒29	121 (25)	91 (14)	68 (27)	92 (70)	47 (69)
Susceptible to RIF and INH	283 (18)	16‒20	4 (0.8)	256 (39)	18 (7.2)	5 (3.8)	0 (0)
Unknown	813	13‒16	69	34	191	314	205

GADR-TB = Group-A drug-resistant TB; SLDR-TB = second-line drug-resistant TB; FLDR-TB = first-line drug-resistant TB; CI = confident interval; NA = not available; RIF = rifampicin; INH = isoniazid.

The resistance rates to BDQ, LZD, MFX, LFX, and AMK were respectively 3.1%, 0.8%, 15.0%, 13.0%, and 12.0%. Drug-resistant rates for BDQ, LZD and AMK showed no notable variation over the years. However, for MFX, the highest rate was observed in 2019 at 30.0%, and it gradually decreased, stabilising around 16.0% in 2023. This phenomenon was mainly attributed to the difference in testing criteria. The drug-resistant rate for CFZ was considerably lower from 2019 to 2022 when a 2.5 CC was used due to a technical error, and the rate increased to 4.8% in 2023 when a 1.0 CC was used (Table 2).

GADR-TB rates were significantly higher in patients resistant to RIF (6.7%), INH (6.8%), or both (25.3%) compared to those susceptible to RIF and INH (1.1%). Similarly, SLDR-TB rates were higher in patients resistant to RIF (13.3%), INH (8.8%), or both (36.1%) than in those susceptible to RIF and INH (1.8%) (Supplementary Table S6).

**Figure 2.**
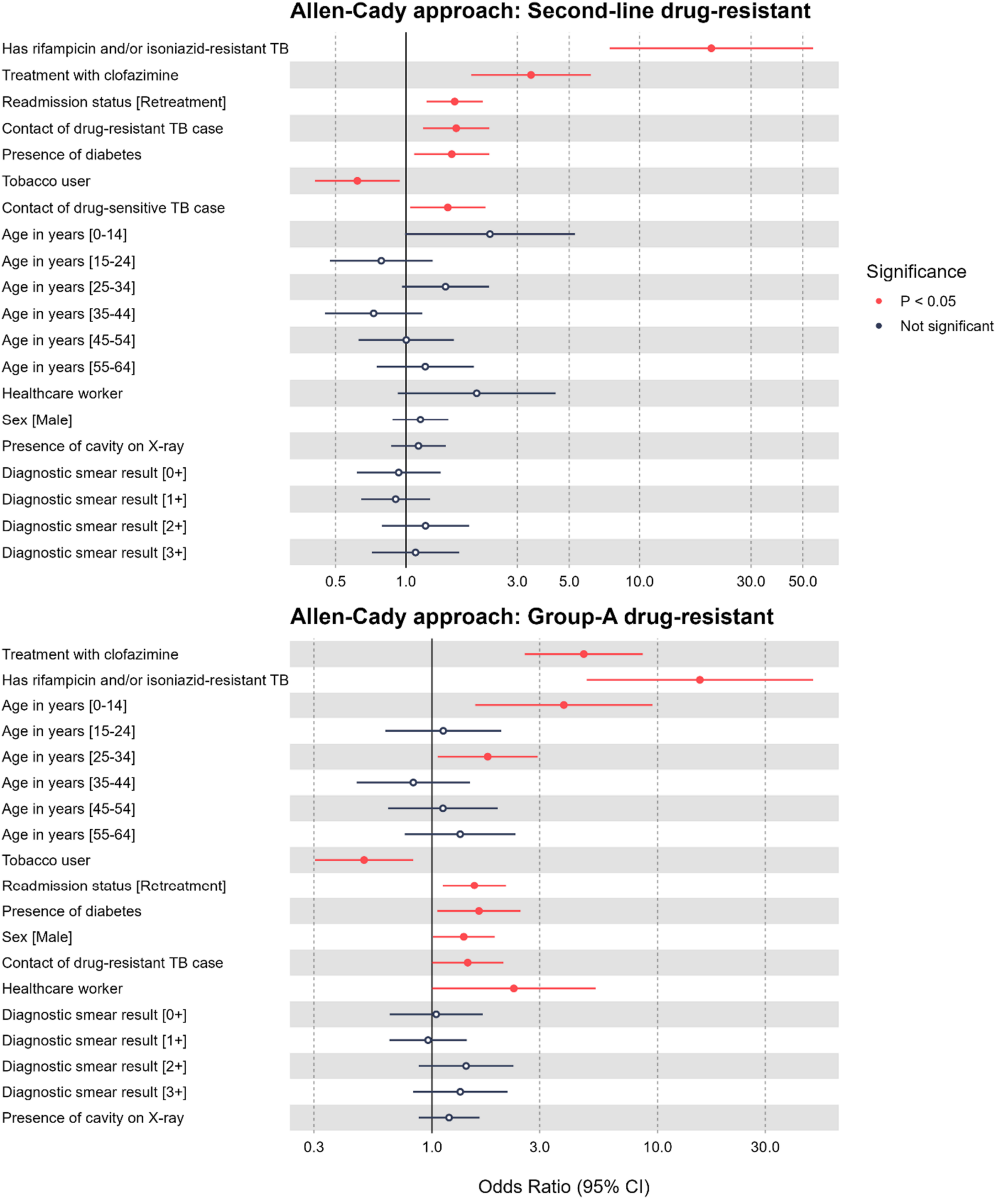
Multivariable analysis of the association between study variables and second-line drug-resistant TB and Group A drug-resistant TB using the Allen-Cady approach for variable selection. For the second-line drug-resistant model, 396 observations were deleted in the final model due to missing values. Variables included in the final model were age category, sex, TB case classification, diagnostic smear, presence of cavity on X-ray, presence of diabetes, treatment history (with ethambutol, rifampicin, pyrazinamide, cycloserine, streptomycin, kanamycin, para-aminosalicylic acid, clofazimine, capreomycin, levofloxacin, and moxifloxacin), contact with drug-susceptible TB patient, contact with drug-resistant TB patient, being a healthcare worker, active or ex-tobacco user, and resistant rifampicin/isoniazid status. For the Group A drug-resistant model, 407 observations were deleted in the final model due to missing values. Variables included in the final model were age category, sex, TB case classification, diagnostic smear, presence of cavity on X-ray, presence of diabetes, treatment history (with ethambutol, rifampicin, pyrazinamide, cycloserine, streptomycin, kanamycin, para-aminosalicylic acid, clofazimine, capreomycin, levofloxacin, and moxifloxacin), contact with drug-resistant TB patient, being a healthcare worker, active or ex-tobacco user, resistant rifampicin/isoniazid status. CI = confidence interval.

The identified risk factors for SLDR-TB and their adjusted odds ratios (aORs) were as follows: resistance to RIF and/or INH (aOR 20.3, 95% CI 8.48–66.3), CFZ exposure (aOR 3.43, 95% CI 1.91–6.25), retreatment cases (aOR 1.62, 95% CI 1.22–2.13), contact with DS-TB case (1.51, 95% CI 1.04–2.18) or DR-TB case (aOR 1.64, 95% CI 1.18–2.27), and DM (aOR 1.57, 95% CI 1.08–2.27). No association was found with age, sex, diagnostic smear results, chest X-ray cavities, or healthcare worker status (Figure 2). Interestingly, active or ex-tobacco users had a negative association with SLDR-TB with an AOR of 0.62 (95% CI 0.40–0.93) (Supplementary Table S2).

Significant risk factors associated with GADR-TB were as follows: age groups 0–14 years (aOR 3.84, 95% CI 1.52–9.37) and 25–34 years (aOR 1.77, 95% CI 1.07–2.98), male (aOR 1.38, 95% CI 1.01–1.90), retreatment cases (aOR 1.54, 1.12–2.12), DM (aOR 1.62, 95% CI 1.05–2.46), CFZ exposure (aOR 4.71, 95% CI 2.59–8.65), contact with DR-TB cases (aOR 1.44, 95% CI 1.00–2.06), and TB resistance to RIF, INH or both (aOR 15.4, 95% CI 5.75–62.8). No associations were found between diagnostic smear results and the presence of cavities in chest X-rays and GADR-TB (Supplementary Table S3). Similarly, active or ex-tobacco users had a negative association with GADR-TB with an AOR of 0.50 (95% CI 0.30–0.81) (Supplementary Table S3).

Sensitivity analysis, conducted using LASSO for variable selection, revealed identical factors to the Allen-Cady approach regarding the association between study variables and SLDR-TB (Supplementary Table S2–S4). However, for GADR-TB, the LASSO approach yielded similar factors, with the exception of the age group 25–34 years and retreatment cases, which were significant in the Allen-Cady approach but not in the LASSO analysis (Supplementary Table S3–S5).

## DISCUSSION

We identified a high prevalence of GADR-TB (16.0%) and SLDR-TB (24.0%) in Karakalpakstan, along with significant risk factors for SLDR-TB. The high prevalence of GADR-TB and SLDR-TB was mainly attributed to FQ resistance, with 62.0% of SLDR-TB and 94.3% of GADR-TB cases exhibiting FQ resistance. This highlights the importance of rapid molecular diagnostics for detecting FQ resistance before commencing TB treatment.

The observed 6.2% prevalence of BDQ resistance among patients with MDR-TB is slightly higher than the rate reported in India (21/1,016, 2.1%),^12^ and China (10/425, 2.4%).^13^ Notably, our 15.0% fluoroquinolone resistance rate is lower than rates reported in India (703/1,016, 69.2%),^12^ China (311/425, 73.2%),^13^ Russia (59/161, 37.0%),^4^ and Ukraine (53/169, 31.4%).^8^ Furthermore, the 0.8% rate of LZD resistance in our study is substantially lower than that reported in India (72/365, 19.7%),^27^ and China (30/425, 7.1%).^13^ These differences may be influenced by contextual and genetic variations in MTB strains, as well as variances in testing criteria.

We identified risk factors associated with GADR-TB and SLDR-TB, including resistance to RIF, INH or both, CFZ exposure, contact with patients with DS-TB or DR-TB, DM, and retreatment cases, which is consistent with findings from other studies.^18^^–20^ Unlike other studies,^18^^,19^ we did not find an association between diagnostic smear and the presence of cavitation on X-ray and SLDR-TB.

We found that the 25–34-year age group was at risk of GADR-TB, consistent with other studies.^19^^,21^ However, the finding that the 0–14 year age group is also at risk of GADR-TB is surprising. Possible explanations include the potential selection bias for MTB diagnosis and pDST in children due to challenges in sample collection. Only children with evidence of treatment failure and contact with patients with DR-TB are prioritised for sputum induction for pDST. Additionally, there is a lack of representation of children younger than 14 years in most studies, leading to limited knowledge about the risk of DR-TB in this age group.

The increased odds of GADR-TB among healthcare workers highlight inadequate infection control measures in healthcare settings. Arne von Delft et al. reported that the risk of healthcare workers’ exposure to TB is a complex phenomenon involving the perception that long-term healthcare workers working in TB institutes are immune to TB and the perception of decreased risk due to habituation to working in high-risk settings.^28^

Finally, we identified negative associations between active or ex-tobacco use and GADR-TB and SLDR-TB, including cigarette smoking and *nasvai* (a type of smokeless tobacco prepared by mixing locally grown tobacco with slaked lime).^29^ While most studies reported tobacco use as an independent risk factor for DR-TB,^18^^,22^ they often focus on cigarette smoking, potentially overlooking the effect of nasvai on DR-TB. Studies have suggested that nasvai increases the risk of oral cancer,^30^ but its effect on SLDR-TB remains unclear. Given our data did not differentiate between cigarette smoking and nasvai, which is more frequently used than tobacco in Uzbekistan,^31^ we hypothesise that the observed negative association may be attributed to nasvai. We recommend future research to explore the association of nasvai with SLDR-TB.

There are limitations to note in our study. First, the retrospective design predisposes the study to missing data and inadequate details on some of the study variables. Secondly, only culture-positive cases were included; hence, the findings may not be representative of all TB cases. Finally, the study was not designed to differentiate between instances of baseline and follow-up resistance.

The main strengths of our study are extensive programmatic data covering a 5-year period, and the study includes pDST for all the major second-line drugs, which are the backbone of RR-TB treatment. Additionally, the sensitivity analysis using the LASSO approach identified consistent risk factors, similar to the Allen-Cady approach, which strengthens the findings of our study.

## CONCLUSIONS

The high prevalence of SLDR-TB and GADR-TB found in this study is a major concern, emphasising the importance of baseline pDST for patients undergoing RR-TB treatment. Risk factors associated with SLDR-TB or GADR-TB include resistance to RIF or INH, exposure to CFZ, retreatment cases, DM, contact with DS-TB or DR-TB patients, male gender, and working as a healthcare worker. These findings could facilitate the early recognition of individuals at risk and help guide clinical practice.

## Supplementary Material


