# Supplementary material for: Second-line drug-resistant TB and associated risk factors in Karakalpakstan, Uzbekistan
Source: IJTLD Open. 2024 Sep 1;1(9):391–7. doi: 10.5588/ijtldopen.24.0351 (PMC11409172; doi:10.5588/ijtldopen.24.0351)
Supplement: Supplementary file 1 [file ijtldopen24-0351_supplementarydata1.docx]

# <http://dx.doi.org/10.5588/ijtldopen.24.0351>

# Second-line drug-resistant TB and associated risk factors in Karakalpakstan, Uzbekistan

**SUPPLEMENTARY DATA**

*Supplementary Figure S1. Diagnosis of pulmonary TB among people >15 years*

*Supplementary Table S1. Baseline characteristics of the study population included in the risk factor analysis*

*Supplementary Table S2. Univariate and multivariable analysis of the association between study variables and second-line drug-resistant tuberculosis using Allen-Cady approach for variable selection*

*Supplementary Table S3. Univariate and multivariable analysis of the association between study variables and group-A drug-resistant tuberculosis using Allen-Cady approach for variable selection*

*Supplementary Table S4. Univariate and multivariable analysis of the association between study variables and second-line drug-resistant tuberculosis using Least Absolute Shrinkage and Selection Operator (LASSO) approach for variable selection*

*Supplementary Table S5. Univariate and multivariable analysis of the association between study variables and group-A drug-resistant tuberculosis using Least Absolute Shrinkage and Selection Operator (LASSO) approach for variable selection*

*Supplementary Table S6. Distribution of phenotypic drug susceptibility testing for bedaquiline, linezolid, moxifloxacin, levofloxacin, amikacin, group-A drug-resistant and second-line drug-resistant by first-line drug-resistant status*

*Supplementary Figure S1****.*** *Diagnosis of pulmonary TB among people aged >15 years*


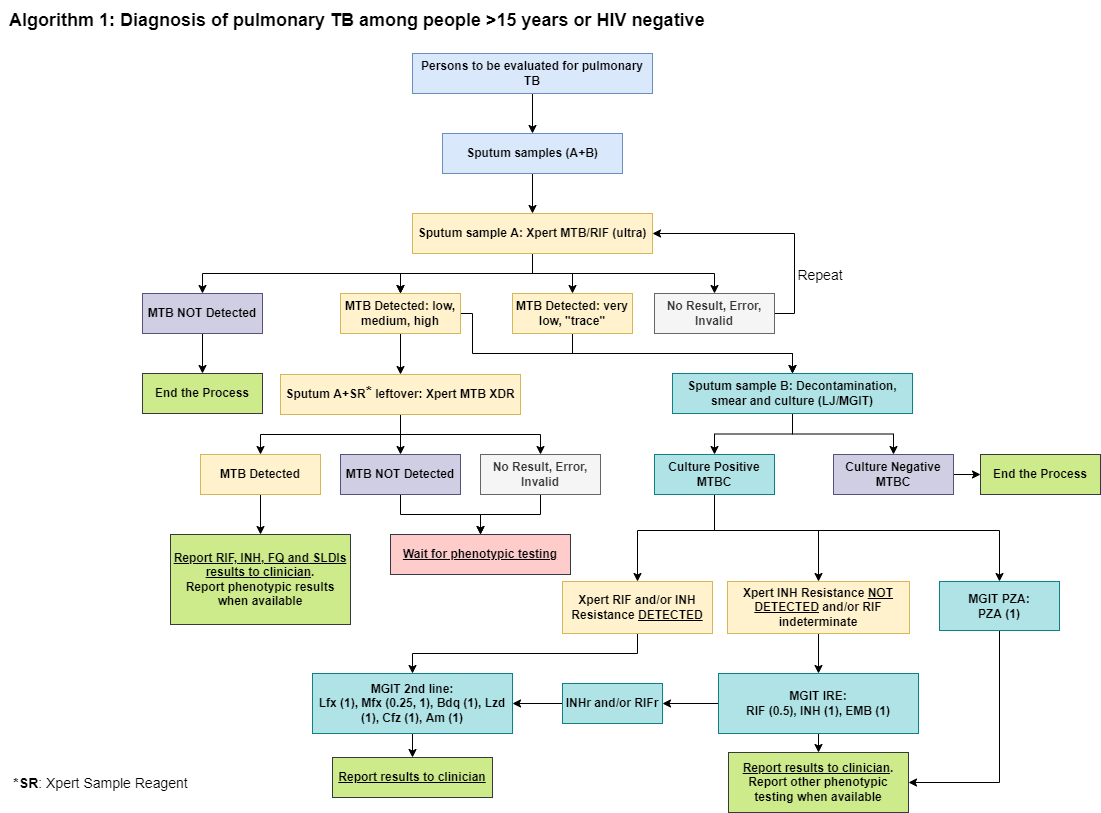


*Acronyms: TB = tuberculosis, MTB = mycobacterium tuberculosis, RIF = rifampicin, INH = isoniazid, PZA = pyrazinamide, FQ = fluoroquinolone, SLDIs = second-line drug injectables, Lfx = levofloxacin, Mfx = moxifloxacin, Bdq = bedaquiline, Lzd = linezolid, Cfz = clofazimine, Am = amikacin, EMB = ethambutol, INHr = isoniazid-resistant, RIFr = rifampicin-resistant.*

*Supplementary Table S1. Baseline characteristics of the study population included in the risk factor analysis*

| **Characteristic** | **N = 1934** |
| --- | --- |
| Age in year | 40 (28, 58) |
| Age category |  |
| 65+ | 322 (17%) |
| 0–14 | 38 (2.0%) |
| 15–24 | 280 (14%) |
| 25–34 | 466 (24%) |
| 35–44 | 306 (16%) |
| 45–54 | 274 (14%) |
| 55–64 | 248 (13%) |
| Sex |  |
| Female | 918 (47%) |
| Male | 1016 (53%) |
| Specimen collection year |  |
| 2019 | 464 (24%) |
| 2020 | 574 (30%) |
| 2021 | 401 (21%) |
| 2022 | 362 (19%) |
| 2023 | 133 (7%) |
| Body mass index | 19.8 (17.7, 22.8) |
| Unknown | 105 |
| Marital status |  |
| Divorced | 95 (5%) |
| Married | 1155 (63%) |
| Separated | 2 (<1%) |
| Single | 370 (20%) |
| Widowed | 207 (11%) |
| Unknown | 105 |
| Employment status |  |
| Disabled | 128 (7%) |
| Employed | 222 (12%) |
| Housework | 6 (<1%) |
| Other | 67 (3.7%) |
| Retired | 419 (23%) |
| Student | 46 (3%) |
| Unemployed | 941 (51%) |
| Unknown | 105 |
| Being a healthcare worker: Yes | 37 (2%) |
| Unknown | 105 |
| History of imprisonment: Yes | 20 (1%) |
| Unknown | 105 |
| History of injecting drug use: Yes | 1 (<1%) |
| Unknown | 106 |
| History of alcohol consumption |  |
| None | 1758 (96%) |
| Moderate | 66 (4%) |
| Excessive | 5 (<1%) |
| Unknown | 105 |
| Active or ex-tobacco user: Yes | 228 (12%) |
| Unknown | 105 |
| History of international travel: Yes | 282 (15%) |
| Unknown | 105 |
| Contact with patient with drug-resistant TB: Yes | 293 (16%) |
| Unknown | 105 |
| Contact with patient with drug-sensitive TB: Yes | 236 (13%) |
| Unknown | 105 |
| Classification of TB cases |  |
| New | 1201 (66%) |
| Retreatment | 628 (34%) |
| Unknown | 105 |
| Had clofazimine exposure for >1month: Yes | 62 (3%) |
| Unknown | 107 |
| Presence of cavity in chest X-ray | 801 (44%) |
| Unknown | 108 |
| People living with HIV: Yes | 8 (<1%) |
| Unknown | 452 |
| Diabetes mellitus: Yes | 256 (14%) |
| Unknown | 105 |
| Diagnostic smear results |  |
| Negative | 776 (43%) |
| 0+ (scanty) | 225 (12%) |
| 1+ | 417 (23%) |
| 2+ | 191 (11%) |
| 3+ | 194 (11%) |
| Unknown | 131 |
| History of exposure to first-line TB drugs: Yes | 651 (36%) |
| Unknown | 107 |
| History of exposure to second-line TB drugs: Yes | 674 (37%) |
| Unknown | 107 |
| History of exposure to group-A TB drugs: Yes | 154 (8%) |
| Unknown | 107 |
| Site of disease |  |
| Pulmonary | 1687 (92%) |
| Extrapulmonary | 142 (8%) |
| Unknown | 105 |
|  | |

*Data are n (%) or median (IQR).* *Acronyms: TB = tuberculosis, IQR = interquartile range.*

*Supplementary Table S2. Univariate and multivariable analysis of the association between study variables and second-line drug-resistant tuberculosis using Allen-Cady approach for variable selection*

*Variables included in the final model were: age category, gender, TB case classification, diagnostic smear, presence of cavity in x-ray, presence of diabetes, treatment history with (ethambutol, rifampicin, pyrazinamide, cycloserine, streptomycin, kanamycin, para-aminosalicylic acid, clofazimine, capreomycin, levofloxacin, and moxifloxacin), contact with drug-sensitive tuberculosis patient, contact with drug-resistant tuberculosis patient, being a healthcare worker, active or ex-tobacco user, resistant rifampicin/isoniazid status. The following variables, including treatment history with (ethambutol, rifampicin, pyrazinamide, cycloserine, streptomycin, kanamycin, para-aminosalicylic acid, capreomycin, levofloxacin, and moxifloxacin), were omitted from the table due to their lack of significant association with second-line drug-resistant tuberculosis and to enhance readability. 396 observations were deleted in the final model due to missingness. Acronyms: TB = tuberculosis, OR = odds ratio.*


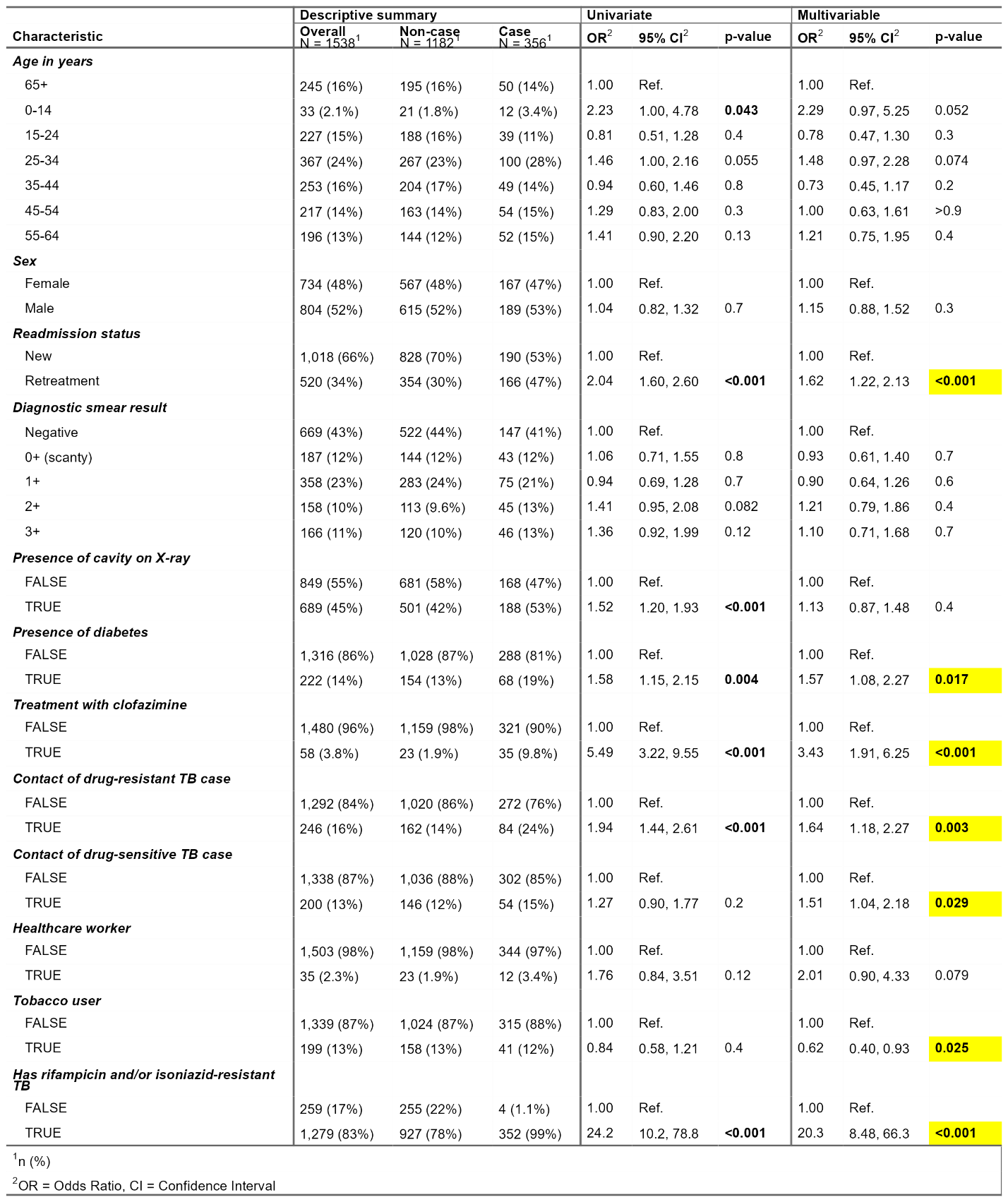


*Supplementary Table S3. Univariate and multivariable analysis of the association between study variables and group-A drug-resistant tuberculosis using Allen-Cady approach for variable selection*

*Variables included in the final model were: age category, gender, TB case classification, diagnostic smear, presence of cavity in x-ray, presence of diabetes, treatment history with (ethambutol, rifampicin, pyrazinamide, cycloserine, streptomycin, kanamycin, para-aminosalicylic acid, clofazimine, capreomycin, levofloxacin, and moxifloxacin), contact with drug-resistant tuberculosis patient, being a healthcare worker, active or ex-tobacco user, resistant rifampicin/isoniazid status. The following variables, including treatment history with (ethambutol, rifampicin, pyrazinamide, cycloserine, streptomycin, kanamycin, para-aminosalicylic acid, capreomycin, levofloxacin, and moxifloxacin), were omitted from the table due to their lack of significant association with second-line drug-resistant tuberculosis and to enhance readability. 407 observations were deleted in the final model due to missingness. Acronyms: TB = tuberculosis, OR = odds ratio.*


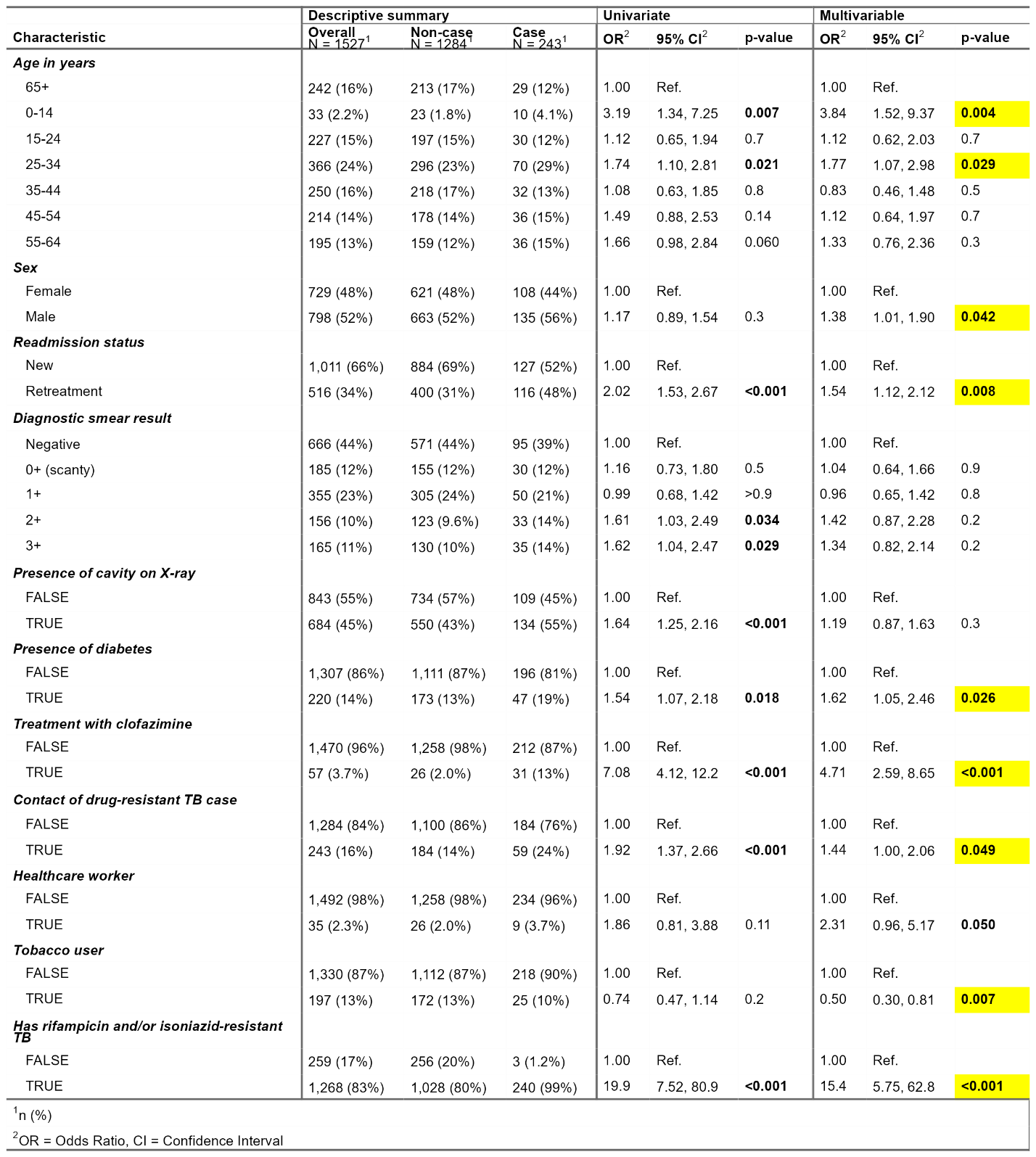


*Supplementary Table S4. Univariate and multivariable analysis of the association between study variables and second-line drug-resistant tuberculosis using Least Absolute Shrinkage and Selection Operator (LASSO) approach for variable selection*

*The automatic variable selection for LASSO method was performed using `{caret}` package in R. The following parameters were set: `method = "repeatedcv"` to resampling method, `number = 5` to indicate the number of sections to split the data into, and `repeats = 10` to indicate the number of times to repeat splitting and testing the model. Variables included in the final model were: age category, resistant rifampicin/isoniazid status, being a healthcare worker, active or ex-tobacco user, contact with drug-resistant tuberculosis patient, contact with drug-sensitive tuberculosis patient, TB case classification, site of the disease, presence of diabetes, treatment history with (cycloserine, clofazimine, and levofloxacin), presence of cavity in x-ray and diagnostic smear. Acronyms: TB = tuberculosis, OR = odds ratio.*


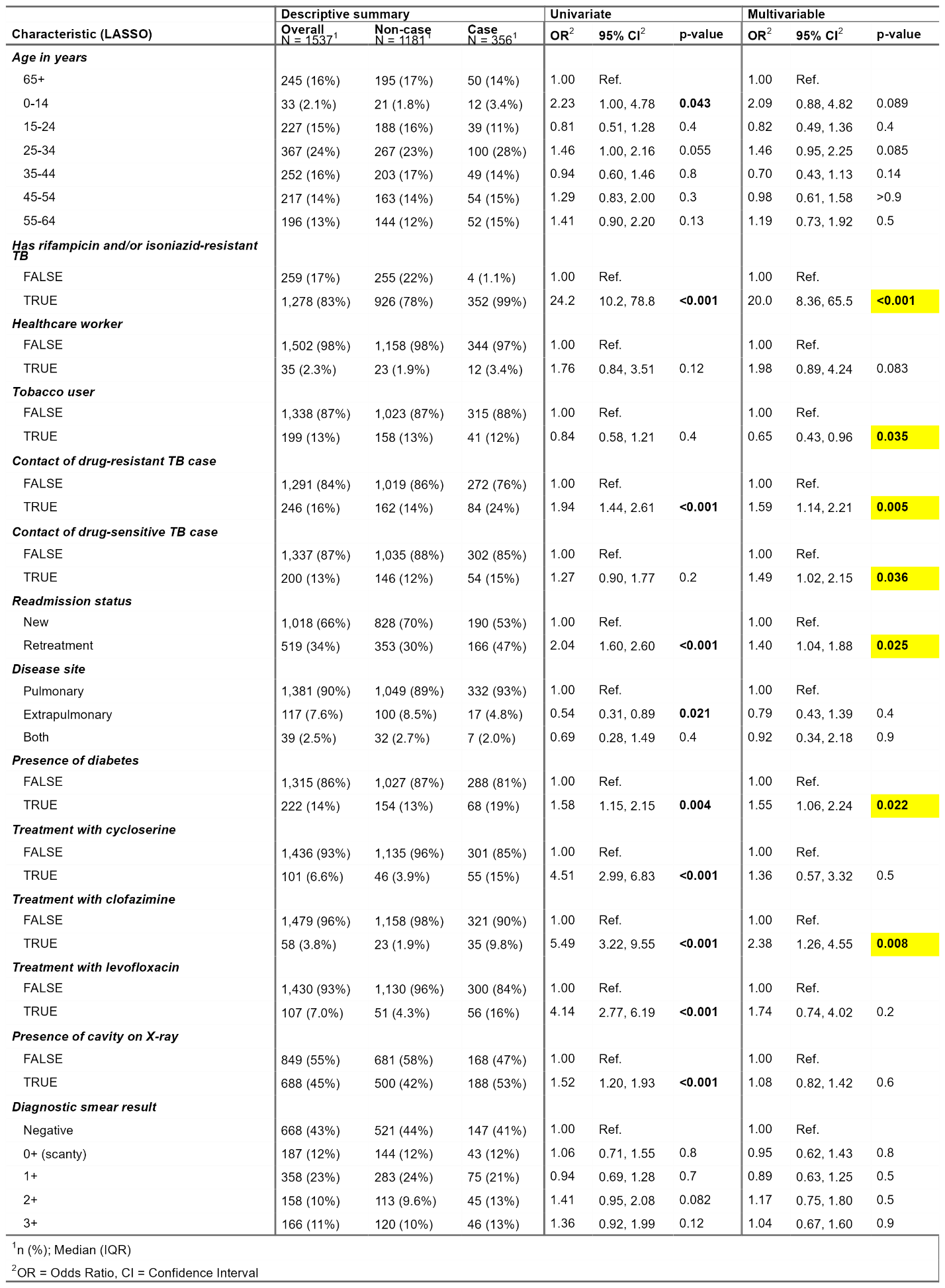


*Supplementary Table S5. Univariate and multivariable analysis of the association between study variables and group-A drug-resistant tuberculosis using Least Absolute Shrinkage and Selection Operator (LASSO) approach for variable selection*

*The automatic variable selection for LASSO method was performed using `{caret}` package in R. The following parameters werset: `method = "repeatedcv"` to resampling method, `number = 5` to indicate the number of sections to split the data into, and `repeats = 10` to indicate the number of times to repeat splitting and testing the model. Variables included in the final model were: age category, gender, resistant rifampicin/isoniazid status, body mass index, being a healthcare worker, imprisonment history, active or ex-tobacco user, migration history, contact with drug-resistant tuberculosis patient, contact with drug-sensitive tuberculosis patient, TB case classification, site of the disease, presence of diabetes, presence of cardiovascular diseases, presence of renal diseases, presence of psychiatric disease, presence of hepatitis diseases, treatment history with (ethambutol, rifampicin, pyrazinamide, cycloserine, streptomycin, kanamycin, para-aminosalicylic acid, clofazimine, capreomycin, levofloxacin, and moxifloxacin), presence of cavity in x-ray and diagnostic smear. Acronyms: TB = tuberculosis, OR = odds ratio.*


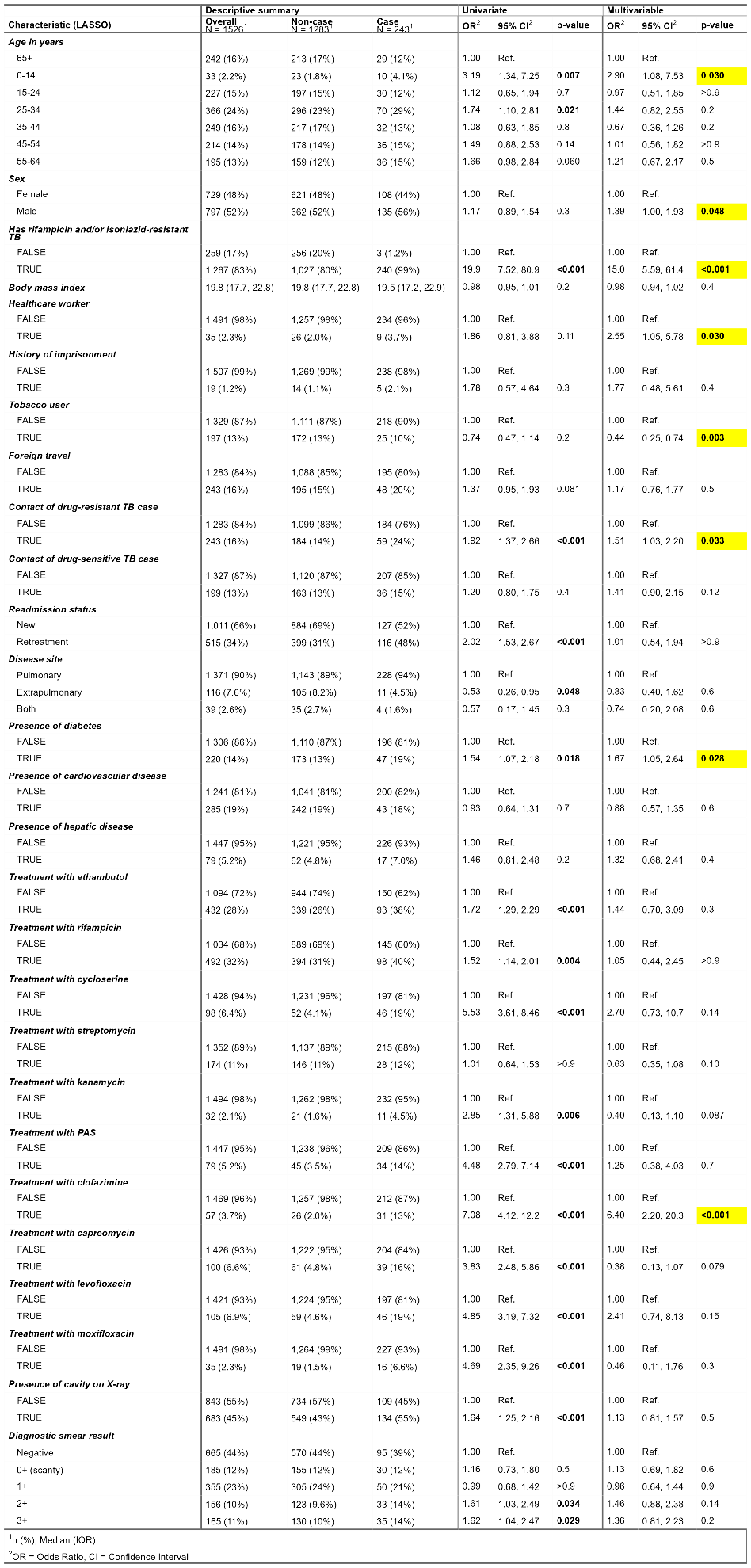


*Supplementary Table S6. Distribution of phenotypic drug susceptibility testing for bedaquiline, linezolid, moxifloxacin, levofloxacin, amikacin, group-A drug-resistant and second-line drug-resistant by first-line drug-resistant status*

| Variable | Overall, N = 1,592*^1^* | Overall, 95% CI | Resistant to RIF & INH, N = 870*^1^* | Resistant to RIF, N = 20*^1^* | Resistant to INH, N = 419*^1^* | Sensitive to RIF & INH, N = 283*^1^* |
| --- | --- | --- | --- | --- | --- | --- |
| Bedaquiline: R | 45 (3.7%) | 2.7%, 5.0% | 37 (6.2%) | 0 (0%) | 6 (1.8%) | 2 (0.7%) |
| S | 1,119 (92%) | 90%, 94% | 535 (90%) | 15 (100%) | 302 (93%) | 267 (95%) |
| I | 51 (4.2%) | 3.2%, 5.5% | 23 (3.9%) | 0 (0%) | 17 (5.2%) | 11 (3.9%) |
| Unknown | 377 |  | 275 | 5 | 94 | 3 |
|  |  |  |  |  |  |  |
| Linezolid: R | 7 (0.6%) | 0.25%, 1.2% | 4 (0.7%) | 0 (0%) | 3 (0.9%) | 0 (0%) |
| S | 1,155 (95%) | 94%, 96% | 565 (95%) | 15 (100%) | 307 (94%) | 268 (96%) |
| I | 53 (4.4%) | 3.3%, 5.7% | 26 (4.4%) | 0 (0%) | 15 (4.6%) | 12 (4.3%) |
| Unknown | 377 |  | 275 | 5 | 94 | 3 |
|  |  |  |  |  |  |  |
| Moxifloxacin: R | 188 (14%) | 12%, 16% | 165 (24%) | 1 (6.7%) | 20 (5.9%) | 2 (0.7%) |
| S | 1,098 (83%) | 81%, 85% | 506 (73%) | 14 (93%) | 306 (90%) | 272 (97%) |
| I | 41 (3.1%) | 2.3%, 4.2% | 21 (3.0%) | 0 (0%) | 14 (4.1%) | 6 (2.1%) |
| Unknown | 265 |  | 178 | 5 | 79 | 3 |
|  |  |  |  |  |  |  |
| Levofloxacin: R | 53 (13%) | 10%, 17% | 42 (22%) | 1 (20%) | 10 (5.0%) | 0 (0%) |
| S | 338 (83%) | 79%, 87% | 139 (74%) | 4 (80%) | 185 (92%) | 10 (91%) |
| I | 14 (3.5%) | 2.0%, 5.9% | 7 (3.7%) | 0 (0%) | 6 (3.0%) | 1 (9.1%) |
| Unknown | 1,187 |  | 682 | 15 | 218 | 272 |
|  |  |  |  |  |  |  |
| Fluoroquinolone: R | 189 (14%) | 12%, 16% | 166 (24%) | 1 (6.7%) | 20 (5.9%) | 2 (0.7%) |
| S | 1,097 (83%) | 80%, 85% | 505 (73%) | 14 (93%) | 306 (90%) | 272 (97%) |
| I | 41 (3.1%) | 2.3%, 4.2% | 21 (3.0%) | 0 (0%) | 14 (4.1%) | 6 (2.1%) |
| Unknown | 265 |  | 178 | 5 | 79 | 3 |
| Clofazimine: R | 14 (1.2%) | 0.66%, 2.0% | 13 (2.2%) | 0 (0%) | 0 (0%) | 1 (0.4%) |
| S | 1,159 (95%) | 94%, 97% | 562 (95%) | 15 (100%) | 311 (96%) | 271 (97%) |
| I | 41 (3.4%) | 2.5%, 4.6% | 19 (3.2%) | 0 (0%) | 14 (4.3%) | 8 (2.9%) |
| Unknown | 378 |  | 276 | 5 | 94 | 3 |
| Amikacin: R | 125 (9.7%) | 8.2%, 11% | 107 (16%) | 1 (6.7%) | 14 (4.2%) | 3 (1.1%) |
| S | 1,121 (87%) | 85%, 89% | 533 (81%) | 14 (93%) | 303 (91%) | 271 (97%) |
| I | 41 (3.2%) | 2.3%, 4.3% | 18 (2.7%) | 0 (0%) | 17 (5.1%) | 6 (2.1%) |
| Unknown | 305 |  | 212 | 5 | 85 | 3 |
| GADR status: R | 202 (15%) | 13%, 17% | 175 (25.3%) | 1 (6.7%) | 23 (6.8%) | 3 (1.1%) |
| S | 1,091 (82%) | 80%, 84% | 501 (72%) | 14 (93%) | 304 (89%) | 272 (97%) |
| I | 35 (2.6%) | 1.9%, 3.7% | 17 (2.5%) | 0 (0%) | 13 (3.8%) | 5 (1.8%) |
| Unknown | 264 |  | 177 | 5 | 79 | 3 |
| SLDR status: R | 287 (22%) | 19%, 24% | 250 (36.1%) | 2 (13.3%) | 30 (8.8%) | 5 (1.8%) |
| S | 1,016 (77%) | 74%, 79% | 433 (62%) | 13 (87%) | 300 (88%) | 270 (96%) |
| I | 25 (1.9%) | 1.2%, 2.8% | 10 (1.4%) | 0 (0%) | 10 (2.9%) | 5 (1.8%) |
| Unknown | 264 |  | 177 | 5 | 79 | 3 |

1 n (%)

Footnote: 813 patients with unknown status for first-line drug-resistant were excluded in the table

Acronym: R = resistant, S = sensitive, I = indeterminate, RIF = rifampicin, INH = isoniazid, GADR = group-A drug-resistant, SLDR = second-line drug-resistant, N = number, CI = confidence Interval.
